# The relationship between hemodialysis mortality and the Chinese medical insurance type

**DOI:** 10.1080/0886022X.2019.1657893

**Published:** 2019-09-05

**Authors:** Xi Yao, Shaohua Chen, Wenhua Lei, Nan Shi, Weiqiang Lin, Xiaoying Du, Ping Zhang, Jianghua Chen

**Affiliations:** a Kidney Disease Center, The First Affiliated Hospital, College of Medicine, Zhejiang University, Hangzhou, Zhejiang, China;; b Key Laboratory of Kidney Disease Prevention and Control Technology, Hangzhou, Zhejiang, China

**Keywords:** End-stage renal disease (ESRD), hemodialysis, medical insurance, mortality

## Abstract

**Objectives:** In China, there are two major medical insurance models: the Urban Basic Medical Insurance (UBMI) and the New Cooperative Medical Scheme (NCMS). The aim of the present study was to evaluate the association of the medical insurance type of patients undergoing hemodialysis (HD) with their survival.

**Methods:** We retrospectively analyzed the end-stage renal disease adult patients initiating HD between January 2010 and December 2014 in Zhejiang province, followed up through 31 December 2015. Patients who had received HD for over 3 months were separated into two groups, based on different medical insurance type. Demographic, clinical data, and clinical outcomes were analyzed. The survival rates were calculated by using the Kaplan–Meier method.

**Results:** A total of 6779 patients (59 ± 16 years old, 4331 males (63.9%)) with UBMI and 7177 (59 ± 16 years old, 3778 males (52.8%)) with NCMS enrolled from 226 hemodialysis units. Compared with UBMI group, patients with NCMS had a smaller percentage of hypertensive nephropathy, diabetes mellitus and arteriovenous fistula, faced with more problems in anemia, hypoalbuminemia and metabolism of calcium and phosphorous. The 1-, 3- and 5-year survival rates were 95.4, 84.4, and 74.1% in UBMI group, 93.1, 79.7, and 67.7% in NCMS group, respectively. Patients with NCMS showed higher all-cause mortality compared with UBMI (*p* < 0.001). In multivariate Cox proportional hazards model, NCMS was independently associated with higher mortality (AHR = 1.53; 95% CI 1.38 ∼ 1.68).

**Conclusions*:*** The medical insurance model was independently associated with HD patient survival, NCMS was associated with increased mortality among patients undergoing maintenance hemodialysis in China.

## Introduction

End-stage renal disease (ESRD) occurs when kidneys are functioning at approximately less than 15% of their normal level and the glomerular filtration rate (GFR) reaches lower than 15 ml/min, requiring renal replacement therapy (RRT), namely hemodialysis (HD), peritoneal dialysis (PD) and kidney transplantation, for survival [1]. The prevalence rate of chronic kidney disease (CKD) in Mainland China is reported to be 10.8% [2,3], and 9.88% in Zhejiang province [4], about 2% develop into ESRD. ESRD becomes a major public health problem with high rates of mortality and significant costs to the health care system [3,5]. Many traditional factors are associated with outcomes of patients with ESRD, such as age, gender, a history of diabetes mellitus (DM) or cardiovascular disease (CVD), types of vascular access, nutritional status and mineral bone disorder [6–11]. Recent researches showed that socioeconomic characteristics may also have a relation to outcomes of dialysis patients [12]; low-income, low education, living in remote or rural areas and lack of social and family’s support was associated with poor survival rate and quality of life in HD patients [13–18].

With the rapid growth of ESRD patients in recent years, the dialysis costs have kept increasing, which have become a heavy burden on families and societies. In 2009, the Chinese government launched a new round of national healthcare system reform [19], providing two major types of medical insurance – the New Cooperative Medical Scheme (NCMS) and the Urban Basic Medical Insurance (UBMI), including the Urban Employee Basic Medical Insurance (UEBMI) and the Urban Residents Basic Medical Insurance (URBMI). Differences exist in the coverage, fund raising and operation of UBMI and NCMS. UBMI is mainly for urban employees, retired citizens, non-employed residents, students and children, and NCMS nearly covers all rural residents [20]. In Zhejiang province, the reimbursement rates of UEBMI, URBMI and NCMS for HD patients with ESRD were approximately 95, 80, and 50 ∼ 80%, respectively. Previous Chinese research has found that Chinese medical insurance had a relation to survival of PD patients, NCMS is independently associated with lower survival [21]. However, no study investigated the association between medical insurance model and the outcome of HD patients in China till now.

The aim of the present study was to assess the association between medical insurance model and outcomes of maintenance hemodialysis (MHD) patients with ESRD from eastern China.

## Methods

### Study design

This is a multicenter, retrospective cohort study, including all incident ESRD patients undergoing MHD between January 2010 and December 2014 from Zhejiang province, a vast province in eastern China. All patients were followed up until death or switching to other RRT, or December 31st, 2015 (the end of the study), after which survival data were censored. Inclusion criteria were: (1) aged 18 years and older; (2) received hemodialysis for more than 90 days; and (3) provided complete information about their medical insurance type and clinical characteristics. Exclusion criteria were: (1) refused to provide written consent; (2) malignant disease; (3) the patient had no insurance or commercial insurance; (4) with previous RRT. This study was performed in accordance with the Declaration of Helsinki and approved by the Research Ethics Committee of the First Affiliated Hospital, College of Medicine, Zhejiang University. The clinical trials registration number is 2018008 and the date of registration is January 2018. All participants provided informed consent before enrollment.

### Patient cohort

Data were obtained from a dialysis registration system - Zhejiang Renal Disease System (ZJRDS) database, which was established in 2007 and included 226 hemodialysis centers all over the Zhejiang province up to present. Patients were separated into UBMI and NCMS group according to different medical insurance model. The earliest records of demographic and clinical data, such as gender, age, etiology of ESRD, BMI (Weight (kilogram/Height^2^ metres^2^)), type of vascular access, and laboratory parameters in the first three months after the beginning of hemodialysis, were extracted from ZJRDS database. Laboratory data was measured by every dialysis units and uploaded with a uniform measurement unit.

### Clinical outcomes

The clinical outcomes were all-cause mortality, switching to PD and kidney transplantation. Causes of death were grouped as: (1) cardiovascular diseases, CVD (congestive heart failure, ischemic heart disease, cardiac valvular disorders, rhythm disturbance, cardiomyopathy, hypertension, peripheral vascular diseases, cerebrovascular disease); (2) infection; (3) systemic failure; (4) financial reasons (gave up treatment because of the family could not afford high medical expenses); (5) other or unknown causes (suicide, traffic accident and so on).

### Statistical analyses

Continuous variables are expressed as mean ± SD for normally distributed data, or as median and frequency (25 ∼ 75%) for non-normally distributed data. Categorical data are presented as proportions. Differences in demographics, clinical characteristics between the two groups were analyzed using the unpaired *t*-test for normally distributed continuous data, Mann–Whitney U test for non-normally distributed data, and chi-square test for categorical data. Laboratory parameters were analyzed by using the standardized mean difference (SMD) and its 95% confidence interval (CI). Survival curves were generated using the Kaplan–Meier method. Mortality hazard ratios (HRs) for different medical insurance (NCMS vs. UBMI) in MHD patients were using multivariate Cox proportional hazards model, adjusted for age, gender, primary diseases, complications and type of vascular access. Statistical analysis was performed using SPSS 22.0 (IBM, Armonk, NY, USA) and Stata software version 12 (StataCorp, College Station, TX). Statistical significance was defined as *p* < 0.05.

## Results

### Demographic and clinical characteristics

**Figure 1.**
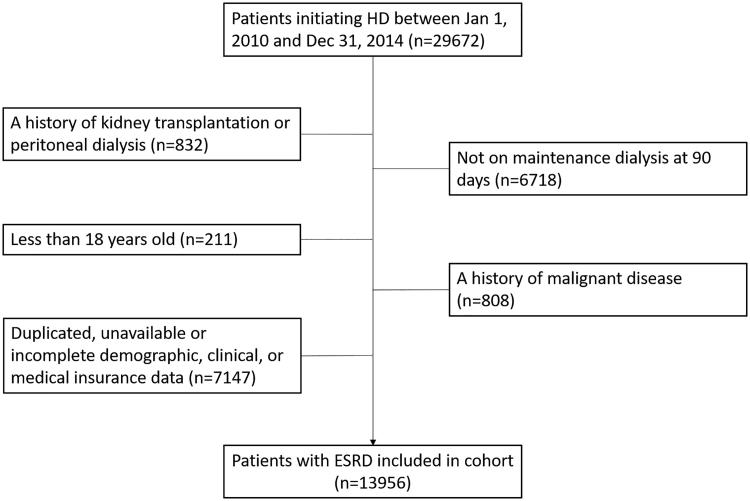
Derivation of cohort.

A total of 29 672 incident dialysis patients were enrolled from 2010 to 2014, and 15 716 patients were excluded. The progress of selecting the whole cohort is shown in Figure 1. The remaining 13 956 patients were included in the final analysis, 48.6% patients (*n* = 6779) with UBMI and 51.4% patients (*n* = 7177) with NCMS; baseline demographic and clinical characteristics are presented in Table 1. Compared with patients with NCMS, patients with UBMI were heavier (body weight: 56.6 vs. 53.5 kg, *p* < 0.001; BMI 20.7 vs. 20.1 kg/m^2^, *p* < 0.001), had a higher percentage of the aged (65+ years old, 42.9 vs. 39.1%, *p* < 0.001), diabetic nephropathy (DN, 23.7 vs. 19.3%, *p* < 0.001), hypertensive nephropathy (HTN, 8.7 vs. 7.6%, *p* < 0.05), diabetes mellitus (DM, 6.6 vs. 5.0%, *p* < 0.001) and arteriovenous fistula (AVF, 26.0 vs. 21.6%, *p* < 0.001), but had a lower proportion of chronic glomerulonephritis (CGN, 45.0 vs. 50.4%, *p* < 0.001). There was no difference in cerebrovascular diseases complication (CVD) between the two groups.

**Table 1.** Demographic and baseline characteristics of the patients.

Characteristics	Patients with UBMI (*n* = 6779)	Patients with NCMS (*n* = 7177)	*p*-Value
Age (year)	59 ± 16	58 ± 15	<0.001
Age groups, *n* (%)			
<65 years	3870 (57.1)	4371 (60.9)	<0.001
65+ years	2909 (42.9)	2806 (39.1)	<0.001
Male, *n* (%)	4331 (63.9)	3778 (52.8)	<0.001
Primary diseases, *n* (%)			
CGN, *n* (%)	3053 (45.0)	3617 (50.4)	<0.001
DN, *n* (%)	1606 (23.7)	1385 (19.3)	<0.001
HTN, *n* (%)	593 (8.7)	542 (7.6)	<0.05
PKD, *n* (%)	289 (4.3)	354 (4.9)	0.059
Other/unknown, *n* (%)	1238 (18.3)	1279 (17.8)	0.498
Complications, *n* (%)			
DM, *n* (%)	446 (6.6)	362 (5.0)	<0.001
CVD, *n* (%)	1416 (20.9)	1591 (22.2)	0.066
Vascular access, *n* (%)			
Arteriovenous fistula, *n* (%)	1760 (26.0)	1552 (21.6)	<0.001
Central venous catheters, *n* (%)	3137 (46.3)	4110 (56.7)	<0.001
Body weight (kg)	56.6 (50.0-63.9)	53.5 (47.2-60.1)	<0.001
BMI (kg/m^2^)	20.7 (18.8-23.0)	20.1 (18.2-22.3)	<0.001

Data are presented as mean ± SD or median and frequency (25 ∼ 75%). The differences between groups were analyzed using unpaired *t*-test for normally distributed continuous data, Mann–Whitney U test for non-normally distributed data, and chi-square test for categorical data. UBMI: the Urban Basic Medical Insurance; NCMS: New Cooperative Medical Scheme; CGN: chronic glomerulonephritis; DN: diabetic nephropathy; HTN: hypertensive nephropathy; PKD: polycystic kidney disease; DM: diabetes mellitus; CVD: cerebrovascular diseases; BMI: body mass index.

### Laboratory parameters

During follow-up, patients with NCMS showed lower hemoglobin levels (97.97 ± 13.47 vs. 94.93 ± 13.76 g/l, *p* < 0.001), serum albumin levels (38.15 ± 4.19 vs. 37.94 ± 4.3 g/l, *p* < 0.05), triglycerides levels (1.91 ± 1.66 vs. 1.76 ± 1.1, *p* < 0.001), calcium levels (2.2 ± 0.19 vs. 2.15 ± 0.21 mmol/l, *p* < 0.001), and higher serum creatinine levels (781.51 vs. 796.21 μmol/l, *p* < 0.001), blood urea nitrogen levels (20.83 vs. 21.07 mmol/l, *p* < 0.05), serum uric acid levels (405.35 vs. 416.88 mmol/l, *p* < 0.001), potassium levels (4.65 vs. 4.74 mmol/l, *p* < 0.001), phosphorus levels (1.65 ± 1.68 mmol/l, *p* < 0.001), parathyroid hormone levels (302.2 ± 282.06 vs. 319.41 ± 281.71 pg/ml, *p* < 0.05), and alkaline phosphatase levels (97.89 ± 69.07 vs. 102.48 ± 71.99, *p* < 0.05), compared with UBMI. There was no difference in ferritin, total cholesterol, low-density lipoprotein, high-density lipoprotein, and C-reactive protein levels between the two groups (Table 2).

**Table 2.** Biochemical characteristics of the study patients.

Characteristics	Patients with UBMI (*n* = 6779)	Patients with NCMS (*n* = 7177)	SMD	95%CI	*p*-Value
HGB (g/l)	97.97 ± 13.47	94.93 ± 13.76	0.22	0.19, 0.26	<0.001
FERR	312.1 ± 299.05	322.38 ± 291.88	−0.04	−0.08, 0.01	0.114
ALB (g/l)	38.15 ± 4.19	37.94 ± 4.3	0.05	0.01, 0.09	<0.05
sCr (μmol/l)	781.51 (605.16 − 947.61)	796.21 (640.12 − 972.93)	−0.10	−0.21, −0.13	<0.001
BUN (mmol/l)	20.83 (17.64 − 24.10)	21.07 (17.64 − 24.53)	−0.06	−0.10, −0.02	<0.05
UA (mmol/l)	405.35 (351.31 − 461.88)	416.88 (365.98 − 471.45)	−0.17	−0.21, −0.13	<0.001
TC (mmol/l)	4.25 ± 1.46	4.26 ± 1.24	−0.01	−0.05, 0.03	0.546
TG (mmol/l)	1.91 ± 1.66	1.76 ± 1.1	0.11	0.07, 0.15	<0.001
LDL (mmol/l)	2.31 ± 1.02	2.29 ± 0.83	−0.14	−0.22, −0.06	0.301
HDL (mmol/l)	1.18 ± 0.77	1.18 ± 0.47	−0.00	−0.04, −0.03	0.920
CRP (mg/l)	12.66 ± 17.78	12.44 ± 18.53	0.01	−0.03, 0.05	0.561
K (mmol/l)	4.64 (4.25 − 5.05)	4.74 (4.31 − 5.17)	−0.13	−0.17, −0.09	<0.001
Ca (mmol/l)	2.2 ± 0.19	2.15 ± 0.21	0.23	0.20, 0.27	<0.001
P (mmol/l)	1.65 (1.39 − 1.95)	1.68 (1.43 − 1.99)	−0.09	−0.13, −0.05	<0.001
PTH (pg/ml)	302.2 ± 282.06	319.41 ± 281.71	−0.06	−0.10, −0.02	<0.05
ALP (U/l)	97.89 ± 69.07	102.48 ± 71.99	−0.07	−0.11, −0.02	<0.05

Data are presented as mean ± SD or median and frequency (25 ∼ 75%). The differences between groups were analyzed using SMD and its 95% CI. UBMI: the Urban Basic Medical Insurance; NCMS: New Cooperative Medical Scheme; HGB: hemoglobin; FERR: ferritin; ALB: albumin; sCr: serum creatinine; BUN: blood urea nitrogen; UA: uric acid; TC: total cholesterol; TG: triglycerides; LDL: Low-density lipoprotein; HDL: high-density lipoprotein; CRP: C-reactive protein; K: potassium; Ca: calcium; P: phosphorus; PTH: parathyroid hormone; ALP: alkaline phosphatase.

### Clinical outcomes

Among enrolled patients, 16.2% patients (*n* = 1099) with UBMI and 20.8% patients (*n* = 1494) with NCMS died before the end of this study. Patients with NCMS had a higher unadjusted death rate on univariate analysis (16.2 vs. 20.8%, *p* < 0.001), had a smaller percentage of kidney transplantation (2.0 vs. 3.5%, *p* < 0.001) and lost (7.4 vs. 10.0%, *p* < 0.001) compared with patients with UBMI. There was no difference in switching to PD rate between the two groups. (Table 3).

**Table 3.** Causes for ceasing hemodialysis.

Characteristics	Patients with UBMI (*n* = 6779)	Patients with NCMS (*n* = 7177)	*p*-Value
Death, *n* (%)	1099 (16.2)	1494 (20.8)	<0.001
Switch to PD, *n* (%)	103 (1.5)	112 (1.6)	0.844
Transplant, *n* (%)	235 (3.5)	146 (2.0)	<0.001
Lost, *n* (%)	676 (10.0)	533 (7.4)	<0.001

The differences between groups were analyzed using chi-square test for categorical data. UBMI: the Urban Basic Medical Insurance; NCMS: New Cooperative Medical Scheme; PD: peritoneal dialysis.

### Patients’ survival and predictors of mortality

**Figure 2.**
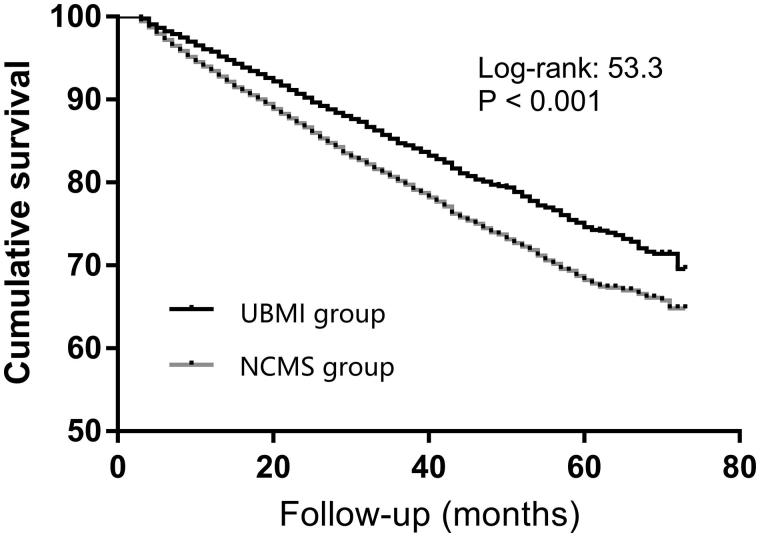
Cumulative survival for patients with UBMI and NCMS.

In the present study, the unadjusted survival rate at 1-, 3- and 5-years were 95.4, 84.4, and 74.1% in patients with UBMI, 93.1, 79.7, and 67.7% in patients with NCMS, respectively (Table 4). Cardiovascular disease was the main cause of death in both groups (Table 5). Kaplan–Meier analysis showed higher survival in patients with UBMI (*p* < 0.001) (Figure 2), and the relative risk of death for patients with NCMS was 1.53 (95% CI, 1.38 ∼ 1.68) in model adjusted for age, sex, primary diseases, complications and type of vascular access. Multivariate Cox proportional regression analysis showed that gender, age, BMI, causes of ESRD, DM, CVD, and vascular access type were independent predictors of patient survival in hemodialysis patients (Table 6).

**Table 4.** Unadjusted hemodialysis patients’ survival rate.

Medical insurance	1-year (%)	3-year (%)	5-year (%)	Median survival time (months)
Patients with UBMI	95.4	84.4	74.1	62
Patients with NCMS	93.1	79.7	67.7	58.9

UBMI: the Urban Basic Medical Insurance; NCMS: New Cooperative Medical Scheme.

**Table 5.** Causes of mortality.

Causes of death	Patients with UBMI (*n* = 1029)	Patients with NCMS (*n* = 1420)	*p*-Value
CVD, *n* (%)	381 (37.0)	467 (32.9)	<0.05
Infection, *n* (%)	131 (12.7)	101 (7.1)	<0.001
Systemic failure, *n* (%)	97 (9.4)	92 (6.5)	<0.05
Financial reasons, *n* (%)	1 (0.1)	10 (0.7)	<0.05
Other, *n* (%)	419 (40.7)	750 (52.8)	<0.001

The differences between groups were analyzed using chi-square test for categorical data. UBMI: the Urban Basic Medical Insurance; NCMS: New Cooperative Medical Scheme; CVD: cardiovascular diseases.

**Table 6.** Predictors of mortality on multivariate Cox proportional regression analysis.

Characteristics	*p*-Value	Adjusted HR	95%CI
NCMS:UBMI	<0.001	1.53	1.38, 1.68
Gender	<0.001	1.21	1.10, 1.34
Age	<0.001	1.05	1.04, 1.05
BMI	<0.001	0.94	0.92, 0.95
Primary Diseases	<0.001		
DN	<0.001	1.98	1.75, 2.23
HTN	<0.05	1.26	1.07, 1.47
PKD	0.111	0.78	0.57, 1.06
Other/Unknown	<0.001	1.29	1.12, 1.48
Complications			
DM	<0.001	1.52	1.26, 1.82
CVD	<0.001	1.39	1.25, 1.54
Vascular access	<0.001		
Arteriovenous fistula	<0.05	0.84	0.72, 0.98
Central venous catheters	<0.001	1.52	1.34, 1.72

Mortality hazard ratios (HRs) for different medical insurance (NCMS vs. UBMI) in maintenance hemodialysis patients using Cox model, adjusted for age, gender, primary diseases, complications and type of vascular access. UBMI: the Urban Basic Medical Insurance; NCMS: New Cooperative Medical Scheme; BMI: body mass index; DN: diabetic nephropathy; HTN: hypertensive nephropathy; PKD: polycystic kidney disease; DM: diabetes mellitus; CVD: cerebrovascular diseases.

## Discussion

The present study was the first multicenter and retrospective analysis of the association between medical insurance type and clinical outcomes of initiated HD patients with ESRD in China. Data showed that patients with NCMS had a smaller percentage of DN, HTN, DM, and AVF, faced with more problems in anemia, hypoalbuminemia, metabolism of calcium and phosphorous, and had a significantly lower survival rate compared with UBMI.

Patients with ESRD have a higher mortality rate than general population, anemia, hypoalbuminemia, infection [22], lower intact parathyroid hormone level [23], upper gastrointestinal bleeding [24], obesity [25], and lower vitamin D [26] are traditional risk factors for mortality in ESRD patients. Meanwhile, socioeconomic factors such as depression [27, 28], education level [13], household income [29] were reported to be associated with survival of ESRD patients. Medical insurance disparity might affect the choice of treatment of patients with ESRD [30], and eventually have an influence on their outcomes. Jurkovitz CT, et al presented that lack of medical insurance is an independent risk factor for early death and ESRD in adults younger than 65 years are at high risk of kidney disease [31]. However, another study showed that the medical insurance status was not independently associated with outcomes in hemodialysis patients [32]. Pieces of evidence about the association between mortality and medical insurance in ESRD patients are controversial, it may relate to a different health insurance policy, economic status, education, nutrition, and technical level of the hospital. In our study, the all-cause mortality was significantly higher in HD patients with NCMS compared with UBMI. Similar results were observed from a previous study from China showing medical insurance model is independently associated with PD patient survival [21], it was also supported by a study from Colombia showing that dialysis patients with poor medical insurance type had lower survival [33].

Currently, China suffers from imbalanced development between rural and urban areas, patients with UBMI mainly live in urban areas, having access to better educational and medical resources, and might consciously contact with a nephrologist and obtain professional medical advice compared with patients with NCMS, who are mainly live in rural areas. The awareness of CKD was low in China [34,35]. Our study showed that the percentage of young patients (<65 years old) in NCMS group was higher than UBMI group, young patients might be related to lower attention to health and awareness of ESRD than the aged, such condition can delay intervention and treatment at an early stage. And unbalanced medical development between rural and urban areas might delay appropriate treatments in rural patients then ultimately allow CKD to progress to ESRD at a young age. Urban patients have more opportunities to receive advises from a nephrologist, who can give suggestions for ESRD patients before starting dialysis, for example, establishing the arteriovenous fistula in advance. Indeed, urban patients had a higher proportion of arteriovenous fistula as vascular access than rural patients in our study, which could be associated with a lower all-cause mortality rate than patients with central venous catheters.

Previous epidemiological data from China showed that patients with UBMI have a higher proportion of hypertension and diabetes. Similar results were observed in our study, more patients with UBMI suffered from DN, HTN, and diabetes compared with NCMS, which might be results of obesity, unhealthy lifestyle and diets caused by rapidly increased economic development in cities. Dialysis patients often suffer many problems, such as anemia, hypoproteinemia, malnutrition, mineral and bone abnormalities. Our study showed that patients with NCMS had lower hemoglobin, serum albumin, and total triglyceride levels than patients with UBMI, which might be a consequence of malnutrition due to a lower economic status in rural areas. Meanwhile, problems in metabolism of calcium and phosphorous were more common in patients with NCMS, who usually are unable to afford expensive phosphorus binders such as lanthanum carbonate, sevelamer hydrochloride and calcimimetics such as cinacalcet hydrochloride, which were not covered by both medical insurance before 2015.

In addition, insurance disparity may affect the choice of treatment for patients with ESRD. Patients with UBMI had more opportunities to receive renal transplantations, although the limitation of the shortage of donor kidneys, financial conditions may play an important part in it. Costs of the living donor nephrectomy are at patients own expense in China, and the reimbursement proportion of follow-up treatment with immunosuppressants is higher in recipients with UBMI than with NCMS. There was no significant difference in the rate of switching from HD to PD between two groups, the exhaustion or infections of the vascular access are thought to be major reasons for this switch [36]. Cardiovascular diseases were major causes of death in hemodialysis patients with ESRD, which proportion was higher in patients with UBMI compared with NCMS; it may be a result of diabetes, hypertension, and obesity common in cities. We followed up patients to tract the reasons of treatment withdrawal and found that the mortality due to financial reasons was still more frequent in patients with NCMS compared with patients with UBMI, underling the importance of insurance coverage and economics in HD patients with ESRD. In addition, results showed that gender, age, DN, HTN, DM, CVD, and central venous catheters were independent predictors for all-cause mortality in hemodialysis patients. We need to make more efforts to manage the controllable factors to reduce the mortality risk.

Our study has several limitations: (1) It is a retrospective study, despite our best efforts to collect some potentially important factors for patients survival, but such as Kt/V, drugs, were unable to be obtained; (2) We did not record the socio-economic status of the enrolled patients such as income and education levels in our database, which may also influence the evaluation of the relationship between medical insurance and mortality in HD patients; (3) We failed to analyze the influence of different communities (rural or urban areas) and different medical insurances (UBMI or NCMS) on patients survival. Because there was a large internal floating population between urban and rural areas, it is hard to separate patients with UBMI or NCMS into urban or rural areas group; (4) The cause of death for many patients should be presumed by the nephrologist, especially for the patients who died at home.

## Conclusions

In summary, our study underlined the importance of Chinese medical insurance type in the survival of hemodialysis patients, survival was lower and laboratory parameters were inferior in HD patients with NCMS compared with UBMI. Results of the present study might provide a rationale for individualization and tailoring of the therapeutic approach, particularly for rural patients. In light of our finding, we suggest that the medical insurance coverage rate for patients with ESRD needs to be improved.

## References

[1] Jones DJ, Butler LT, Harris JP, et al. Latent learning in end stage renal disease (ESRD). Physiol Behav. 2015;142:42–47. 25637651 10.1016/j.physbeh.2015.01.033

[2] Zhang L, Wang F, Wang L, et al. Prevalence of chronic kidney disease in China: a cross-sectional survey. Lancet. 2012;379:815–822. 22386035 10.1016/S0140-6736(12)60033-6

[3] Liu Z Nephrology in China. Nat Rev Nephrol. 2013;9:523–528. 23877587 10.1038/nrneph.2013.146

[4] Lin B, Shao L, Luo Q, et al. Prevalence of chronic kidney disease and its association with metabolic diseases: a cross-sectional survey in Zhejiang province, Eastern China. BMC Nephrol. 2014;15:36. 24559433 10.1186/1471-2369-15-36 PMC3936864

[5] Sharma S, Sarnak MJ Epidemiology: the global burden of reduced GFR: ESRD, CVD and mortality. Nat Rev Nephrol. 2017;13:447–448. 28626221 10.1038/nrneph.2017.84

[6] Ma L, Zhao S Risk factors for mortality in patients undergoing hemodialysis: a systematic review and meta-analysis. Int J Cardiol. 2017;238:151–158. 28341375 10.1016/j.ijcard.2017.02.095

[7] Wang M, Obi Y, Streja E, et al. Association of parameters of mineral bone disorder with mortality in patients on hemodialysis according to level of residual kidney function. CJASN. 2017;12:1118–1127. 28487345 10.2215/CJN.11931116 PMC5498357

[8] Ravani P, Quinn R, Oliver M, et al. Examining the association between hemodialysis access type and mortality: the role of access complications. CJASN. 2017;12:955–964. 28522650 10.2215/CJN.12181116 PMC5460718

[9] Kim T, Streja E, Soohoo M, et al. Serum Ferritin Variations and Mortality in Incident Hemodialysis Patients. Am J Nephrol. 2017;46:120–130. 28704813 10.1159/000478735 PMC5926176

[10] Lacson E Jr., Wang W, Lazarus JM, et al. Change in vascular access and mortality in maintenance hemodialysis patients. Am J Kidney Dis. 2009;54:912–921. 19748717 10.1053/j.ajkd.2009.07.008

[11] Zhao X, Wang M, Zuo L Early mortality risk in incident Chinese hemodialysis patients: a retrospective cohort study. Ren Fail. 2017;39:526–532. 28635363 10.1080/0886022X.2017.1337583 PMC6014524

[12] Krishnasamy R, Gray NA Low socioeconomic status adversely effects dialysis survival in Australia. Nephrology (Carlton). 2018;23:453–460. 28383177 10.1111/nep.13053

[13] Huang WH, Lin JL, Lin-Tan DT, et al. Education level is associated with mortality in male patients undergoing maintenance hemodialysis. Blood Purif. 2013;35:316–326. 23920269 10.1159/000351613

[14] Imanishi Y, Fukuma S, Karaboyas A, et al. Associations of employment status and educational levels with mortality and hospitalization in the dialysis outcomes and practice patterns study in Japan. PLOS One. 2017;12:e0170731. 28264035 10.1371/journal.pone.0170731 PMC5338767

[15] Marinovich S, Lavorato C, Rosa-Diez G, et al. The lack of income is associated with reduced survival in chronic haemodialysis. Nefrologia. 2012;32:79–88. 22294006 10.3265/Nefrologia.pre2011.Nov.11110

[16] Thompson S, Gill J, Wang X, et al. Higher mortality among remote compared to rural or urban dwelling hemodialysis patients in the United States. Kidney Int. 2012;82:352–359. 22592186 10.1038/ki.2012.167

[17] Gray NA, Dent H, McDonald SP Renal replacement therapy in rural and urban Australia. Nephrol Dial Transplant. 2012;27:2069–2076. 21984553 10.1093/ndt/gfr584

[18] Huang B, Lai B, Xu L, et al. Low employment and low willingness of being reemployed in Chinese working-age maintained hemodialysis patients. Ren Fail. 2017;39:607–612. 28805490 10.1080/0886022X.2017.1361834 PMC6446148

[19] Yip WC, Hsiao WC, Chen W, et al Early appraisal of China’s huge and complex health-care reforms. Lancet. 2012;379:833–842. 22386036 10.1016/S0140-6736(11)61880-1

[20] National Health and Family Planning Commission of the People’s Republic of China China statistical yearbook of health and family planning. Beijing (China): Peking Union Medical College Press; 2017.

[21] Wang Z, Zhang Y, Xiong F, et al. Association between medical insurance type and survival in patients undergoing peritoneal dialysis. BMC Nephrol. 2015;16:33. 25880687 10.1186/s12882-015-0023-7 PMC4378355

[22] Pecoits-Filho R, Yabumoto FM, Campos LG, et al. Peritonitis as a risk factor for long-term cardiovascular mortality in peritoneal dialysis patients: the case of a friendly fire? Nephrology (Carlton). 2018;23:253–258. 28010053 10.1111/nep.12986

[23] Lee SA, Lee MJ, Ryu GW, et al. Low serum intact parathyroid hormone level is an independent risk factor for overall mortality and major adverse cardiac and cerebrovascular events in incident dialysis patients. Osteoporos Int. 2016;27:2717–2726. 27216997 10.1007/s00198-016-3636-1

[24] Liang CC, Chou CY, Chang CT, et al. Upper gastrointestinal bleeding as a risk factor for dialysis and all-cause mortality: a cohort study of chronic kidney disease patients in Taiwan. BMJ Open. 2016;6:e010439. 10.1136/bmjopen-2015-010439 PMC4861130 27150184

[25] Segall L, Moscalu M, Hogaş S, et al. Protein-energy wasting, as well as overweight and obesity, is a long-term risk factor for mortality in chronic hemodialysis patients. Int Urol Nephrol. 2014;46:615–621. 24474221 10.1007/s11255-014-0650-0

[26] Al-Aly Z Vitamin D as a novel nontraditional risk factor for mortality in hemodialysis patients: the need for randomized trials. Kidney Int. 2007;72:909–911. 17914416 10.1038/sj.ki.5002544

[27] Kimmel PL Depression as a mortality risk factor in hemodialysis patients. Int J Artif Organs. 1992;15:697–700. 1493943

[28] Fan L, Sarnak MJ, Tighiouart H, et al. Depression and all-cause mortality in hemodialysis patients. Am J Nephrol. 2014;40:12–18. 24969267 10.1159/000363539 PMC4128686

[29] Kimmel PL, Fwu C-W, Eggers PW Eggers PW: Segregation, income disparities, and survival in hemodialysis patients. J Am Soc Nephrol. 2013;24:293–301. 23334394 10.1681/ASN.2012070659 PMC3559484

[30] Johansen KL, Zhang R, Huang Y, et al. Association of race and insurance type with delayed assessment for kidney transplantation among patients initiating dialysis in the United States. CJASN. 2012;7:1490–1497. 22837273 10.2215/CJN.13151211 PMC3430955

[31] Jurkovitz CT, Li S, Norris KC, et al. Association between lack of health insurance and risk of death and ESRD: results from the Kidney Early Evaluation Program (KEEP). Am J Kidney Dis. 2013;61:S24–S32. 23507267 10.1053/j.ajkd.2012.12.015 PMC3739048

[32] Siracuse JJ, Gill HL, Epelboym I, et al. Effect of race and insurance status on outcomes after vascular access placement for hemodialysis. Ann Vasc Surg. 2014;28:964–969. 24370501 10.1016/j.avsg.2013.10.016

[33] Sanabria M, Munoz J, Trillos C, et al. Dialysis outcomes in Colombia (DOC) study: a comparison of patient survival on peritoneal dialysis vs hemodialysis in Colombia. Kidney Int Suppl. 2008;73:S165–S172. 10.1038/sj.ki.5002619 18379541

[34] Ene-Iordache B, Perico N, Bikbov B, et al. Chronic kidney disease and cardiovascular risk in six regions of the world (ISN-KDDC): a cross-sectional study. Lancet Glob Health. 2016;4:e307–e319. 27102194 10.1016/S2214-109X(16)00071-1

[35] Wang F, Zhang L, Wang H China National Survey of CKDWG: awareness of CKD in China: a national cross-sectional survey. Am J Kidney Dis. 2014;63:1068–1070. 10.1053/j.ajkd.2014.01.012 24576417

[36] Rocha PN, Sallenave M, Casqueiro V, et al. [Reason for “choosing” peritoneal dialysis: exhaustion of vascular access for hemodialysis?]. J Bras Nefrol. 2010;32:21–26. Portuguese. 21448515

